# The relationship between total cholesterol and postpartum impaired glucose tolerance in women with gestational diabetes mellitus

**DOI:** 10.1186/s12944-020-01316-5

**Published:** 2020-06-18

**Authors:** Dongyu Wang, Wenjing Ding, Shuqia Xu, Haitian Chen, Bin Liu, Zilian Wang

**Affiliations:** 1 Department of Obstetrics and Gynecology, The First Affiliated Hospital of Sun Yat-sen University, 58th, Zhongshan 2nd Road, Guangzhou, 510080 Guangdong China; 2 Department of Plastic and Reconstructive Surgery, The First Affiliated Hospital of Sun Yat-sen University, 58th, Zhongshan 2nd Road, Guangzhou, 510080 Guangdong China

**Keywords:** Gestational diabetes mellitus, Cholesterol, Impaired glucose tolerance, Type 2 diabetes, Postpartum

## Abstract

**Background:**

History of gestational diabetes mellitus (GDM) and serum lipid abnormalities were associated with postpartum impaired glucose tolerance*.* To investigate the association between concentration of total cholesterol (TC), at the time of GDM diagnosis, and risk of postpartum glucose intolerance.

**Methods:**

Women who were diagnosed GDM with a live singleton delivery between January 1, 2013 and December 31, 2017 were included. Women were grouped based on the TC quartiles at the time of GDM diagnosis and had an OGTT at 6–12 weeks after delivery. The relationship between TC and the risk of postpartum glucose intolerance was assessed by COX regression.

**Results:**

A total of 845 women were in the final analysis. Higher TC quartile at diagnosis of GDM was associated with a decreased risk of postpartum glucose intolerance. Women in the highest TC quartile (>7.0 mmol L^− 1^) had approximately only half-risk of any postpartum glucose intolerance, compared with women in the lowest TC quartile (<5.5 mmol L^− 1^).

**Conclusions:**

The decreased concentration of TC, at the time of GDM diagnosis, was related to an increased risk of postpartum abnormal glucose regulation in GDM women. Therefore, because both excessively increased and decreased TC were associated with pregnancy and postpartum complications, the optimal concentration of maternal TC throughout pregnancy remained to be further researched.

## Introduction

Gestational diabetes mellitus (GDM), which is defined as glucose intolerance that develops or is first detected during pregnancy, is a common complication of pregnancy, affecting 17.8% of pregnancies worldwide per year [1]. Glucose homeostasis, in most women, recovers to normal nonpregnancy levels after delivery. However, women with a history of GDM have an 84% increased risk for GDM in future pregnancies [2] and an approximately 7-fold higher risk for developing type 2 diabetes in the future [3] than women without GDM. Although all women with GDM are suggested to undergo a glucose tolerance test 6–12 weeks after delivery, the rates of attendance are still low [4]. Early diagnosis of prediabetes, including impaired fasting glucose (IFG) and impaired glucose tolerance (IGT), can provide an opportunity to reduce the incidence of type 2 diabetes through the implementation of appropriate lifestyle changes, resulting in a decrease in healthcare and economic costs associated with type 2 diabetes. Therefore, early identification of biomarkers associated with high risks of type 2 diabetes can be conducive to promoting health and preventing diseases.

A great deal of research has shown that serum lipid abnormalities are associated with disturbances in glucose metabolism. Cholesterol is one of the most important components of serum lipids, but only a few studies have focused on total cholesterol (TC). Cholesterol is an essential constitutional component of all animal cell membranes and acts as a precursor to many hormones, such as vitamin D, bile acids and steroid hormones [5]. Maternal cholesterol in the plasma membrane is critical for embryo implantation and uteroplacental vascularization [6]. Serum cholesterol decreases in early pregnancy and increases in later pregnancy due to increasing fetal cell formation and placental steroid hormone production [7]. TC consists of cholesteryl ester and free cholesterol. Cholesterol is transported within lipoproteins in the bloodstream, mainly including low-density lipoprotein (LDL) and high-density lipoprotein (HDL). Previous studies have suggested that altered concentrations of lipoprotein have been associated with gestational disorders, such as preeclampsia and GDM [8, 9]. A large retrospective study in China showed that the TC level during early pregnancy was an independent risk factor for GDM [8]. In addition, a previous study reported that a high intake of cholesterol was associated with an increased risk of GDM and type 2 diabetes [10]. In addition, decreased HDL cholesterol (HDL-c) and elevated LDL cholesterol (LDL-c) are commonly considered atherogenic factors and predictors of cardiovascular diseases [11, 12]. Decreased HDL-c was also identified as one of the best predictors of type 2 diabetes development after GDM [13]. However, data on the association between TC during pregnancy and postpartum disturbances in glucose metabolism in women with recent GDM are not widely available.

Thus, a retrospective cohort study of women with GDM was performed to investigate the association between concentrations of TC, measured at the time of the oral glucose tolerance test (OGTT), and the risk of postpartum impaired glucose tolerance, including IFG, IGT and type 2 diabetes.

## Materials and methods

### Study population and clinical data

This study was performed as part of an ongoing cohort study of pregnant women who received antenatal care at one of the largest regional university hospitals in South China (The First Affiliated Hospital of Sun Yat-sen University).

Women who were diagnosed with GDM following an OGTT that was performed between 24 and 28 weeks of gestation were recruited. Only ethnically Chinese women with a live singleton delivery between January 1, 2013 and December 31, 2017 were included in this study. A total of 2839 patients were eligible for this study. Women were excluded if they met the following conditions: established type 1 or type 2 diabetes, pregestational diabetes mellitus (PGDM) diagnosed by the OGTT during pregnancy, primary renal disorders such as nephrotic syndrome, untreated endocrine disorders such as hyperthyroidism and hypothyroidism, use of any drugs that may affect glucose and lipid metabolism, and confirmed hyperlipidemia or metabolic syndromes before pregnancy.

This study was approved by the Institutional Review Board of The First Affiliated Hospital of Sun Yat-sen University (No. [2014]93). All procedures were conducted in accordance with the Declaration of Helsinki.

### Data collection

The following data were collected from medical records: 1) maternal demographics [maternal age, and prepregnancy body mass index (BMI)], 2) medical and obstetric histories (gravidity, parity, history of spontaneous abortion and history of adverse pregnancy, including intrauterine fetal demise, neonatal death, and fetal with chromosome abnormality), 3) pregnancy and postpartum information [the concentration of TC at GDM diagnosis, GDM treatment, preterm birth (PTB), gestational age at delivery, mode of delivery, weight and length of neonates, timing of postpartum OGTT, and results of postpartum OGTT, including normal blood glucose tolerance, IFG, IGT and type 2 diabetes].

### Measures

At the time of the OGTT, blood samples were withdrawn from the antecubital vein after at least 8 h of fasting, and an additional sample for measuring lipid profile was taken at the time fasting blood glucose was obtained. All samples were measured in the laboratory of the Department of Biochemistry of the First Affiliated Hospital of Sun Yat-sen University. Blood glucose and TC were measured with standard enzymatic procedures on an automatic chemistry analyzer (Abbott Aeroset, Chicago, IL, USA).

### Definitions

#### GDM

According to the 2014 Chinese Medical Association diagnostic criteria, GDM was diagnosed when any serum glucose value equaled or exceeded the thresholds during the OGTT, which was performed between 24 and 28 weeks of gestation: fasting blood glucose (FBG), 5.1 mmol L^− 1^; 1 h, 10.0 mmol L^− 1^; and 2 h, 8.5 mmol L^− 1^. However, FBG ≥ 7.0 mmol L^− 1^ or 2-h value ≥11.1 mmol L^− 1^ was considered PGDM, and subjects with PGDM were excluded from our study [14]. All included women diagnosed with GDM underwent lifestyle interventions, including exercise and diet control, to manage blood glucose levels. Insulin treatment was initiated in cases of poor glycemic control. The targeted serum glucose levels were 3.3–5.3 mmol L^− 1^ and 4.4–6.7 mmol L^− 1^ at fasting and 2 h postprandial, respectively.

#### Type 2 diabetes

All women with GDM were advised to undergo another OGTT 6–12 weeks after delivery. Based on the type 2 diabetes guidelines of the Chinese Diabetes Society (2017 edition) [15], women were considered to have IFG if their FBG was ≥6.1 mmol L^− 1^ and < 7.0 mmol L^− 1^ and their 2-h BG was < 7.8 mmol L^− 1^. Women with FBG < 7.0 mmol L^− 1^ and 2-h BG ≥7.8 mmol L^− 1^ and < 11.1 mmol L^− 1^ were categorized as having IGT, and women with FBG ≥7.0 mmol L^− 1^ and 2-h BG ≥11.1 mmol L^− 1^ were categorized as having type 2 diabetes. Women were considered to have normal glucose levels if their FBG was < 6.1 mmol L^− 1^ and 2-h BG was < 7.8 mmol L^− 1^.

#### PTB

PTB was defined as gestational age at delivery less than 37 weeks [16].

#### BMI

Prepregnancy BMI was calculated by dividing the prepregnancy body weight (kilograms) by the square of height (meters).

### Statistical analysis

Data analysis was performed by SPSS 20.0 (Inc., Chicago, IL, USA). Nonnormally distributed variables are presented as medians with interquartile ranges (IQRs), and categorical variables are presented as numbers and percentages. To eliminate the possible effects of outliers on the association between TC and the incidence of postpartum glucose intolerance, we excluded women whose TC levels at the time of GDM diagnosis were identified as outliers.

Cox regression analysis was conducted to assess the association between increasing TC quartile and risk of postpartum glucose intolerance, including IFG, IGT and type 2 diabetes. The outcomes of IGT, type 2 diabetes, and any postpartum disorders of glucose metabolism were assessed separately. In this study, the number of cases of IFG was too small (3 cases) to assess the association. Women were classified into four groups based on TC quartiles, which were calculated according to the distribution of TC among all eligible women regardless of whether they underwent postpartum OGTT. TC quartile values were distributed from 1 to 4 in ascending order. Then, TC quartiles were put into the Cox regression model as a categorical variable, and the first quartile was defined as the reference to measure the trend of the risk of postpartum disturbances in glucose metabolism with the increase in TC quartiles. The time scale was determined as weeks between delivery and time of postpartum OGTT. Unadjusted and adjusted hazard ratios (HRs) are shown with 95% confidence intervals (CIs). A *P* value < 0.05 was considered statistically significant.

## Results

**Fig. 1:**
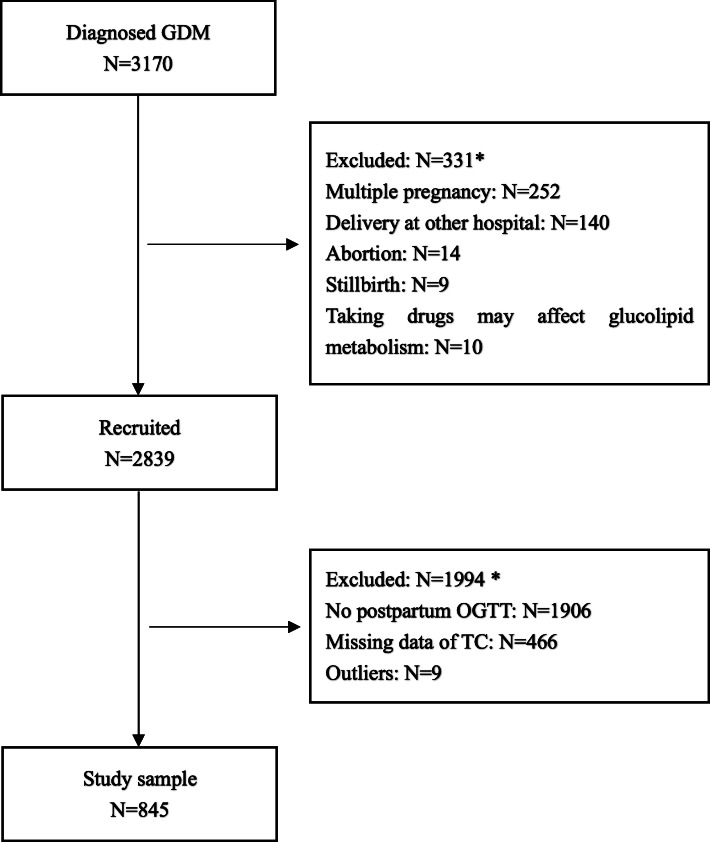
Flow chart for recruitment of 845 women. GDM, gestational diabetes mellitus; OGTT, oral glucose tolerance test; TC, total cholesterol. * There are women who met more than one of the exclusion criteria

A total of 2839 pregnant women met the eligibility criteria, and 845 were included in the final analysis. The detailed screening procedure is shown in Fig. 1. The overall adherence rate for postpartum OGTT in the study population was 30.1%. The clinical data divided by the TC quartiles at GDM diagnosis are listed in Table 1. Glycemic control was ideal in most subjects as a result of lifestyle interventions, but 8 women needed insulin treatment. The concentration of TC at GDM diagnosis ranged from 3.4 mmol L^− 1^ to 9.2 mmol L^− 1^. The prepregnancy BMI of women in the first TC quartile was significantly higher than that of women in the higher TC quartile. The incidence of cesarean delivery in the first TC quartile (28.9%) was significantly higher than that in women in the third (25.6%) and fourth quartiles (21.8%).

**Table 1:** Clinical Data of The Study Population by TC Quartiles at Gestational Diabetes Diagnosis*

	Total	TC Quartiles at GDM diagnosis			
	(IQR) *N* = 845	First (3.4, 5.5) *N* = 212	Second (5.6, 6.2) *N* = 202	Third (6.3, 7.0) *N* = 226	Four (7.1, 9.2) *N* = 205
Demographic data					
Maternal age (year)	33.0 (6.0)	34.0 (7.3)	32.0 (6.0)	32.5 (6.0)	33.0 (8.0)
GA at OGTT (week)	25.14 (1.57)	25.14 (1.85)	25.14 (1.57)	25.29 (1.43)	25.14 (1.57)
Pre-pregnancy BMI (kg m^−2^)	21.37 (3.91)	22.27 (4.06) ^a, b, c^	21.48 (3.79)	21.14 (3.79)	20.96 (3.96)
GWG (kg)	11.00 (5.20)	10.20 (5.50)	11.00 (5.00)	11.00 (5.08)	11.00 (4.50)
TC (mmol l^−1^)	6.30 (1.50)	4.00 (1.80)	6.00 (1.30)	6.60 (0.40)	7.60 (0.80)
TG (mmol l^−1^)	2.21 (1.05)	2.11 (1.03)^c^	2.21 (1.10)	2.25 (1.12)	2.36 (1.00)
LDL-c (mmol l^−1^)	1.96 (0.50)	1.73 (0.40) ^a, b, c^	1.88 (0.39) ^d, e^	2.05 (0.46) ^f^	2.23 (0.45)
HDL-c (mmol l^−1^)	3.51 (1.02)	2.66 (0.57) ^a, b, c^	3.28 (0.36) ^d, e^	3.75 (0.38) ^f^	4.37 (0.66)
Medical and obstetric history					
Primipara (%)	498 (58.9)	119 (23.9)	122 (24.5)	139 (27.9)	118 (23.7)
Spontaneous abortion (%)	146 (17.3)	45 (30.8)	36 (24.7)	31 (21.2)	34 (23.3)
Adverse pregnancy outcomes (%)	17 (2.0)	5 (29.5)	6 (35.3)	3 (17.6)	3 (17.6)
Pregnant and postpartum data					
PTB (%)	89 (10.5)	31 (34.8)	21 (23.6)	18 (20.2)	19 (21.3)
GA at delivery (weeks)	38.71 (1.57)	38.64 (1.71)	38.86 (1.60)	38.86 (1.57)	38.79 (1.43)
Caesarean delivery (%)	450 (53.2)	130 (28.9) ^b, c^	107 (23.8)	115 (25.6)	98 (21.8)
Neonatal weight (kg)	3.12 (0.54)	3.11 (0.63)	3.10 (0.56)	3.15 (0.50)	3.10 (0.42)
postpartum OGTT timing (weeks)	7.57 (1.82)	7.43 (1.85)	7.43 (1.32)	7.71 (2.04)	7.50 (1.71)
Postpartum OGTT (mmol l^−1^)					
Fasting	4.8 (0.6)	4.8 (0.6)	4.8 (0.5) ^e^	4.8 (0.7)	4.9 (0.6)
2-h	7.1 (2.2)	6.4 (1.3) ^b, c^	6.4 (1.5) ^d, e^	7.9 (2.7) ^f^	8.6 (1.2)
IFG (%)	3 (0.4)	1 (33.3)	0	1 (33.3)	1 (33.3)
IGT (%)	267 (31.6)	83 (31.1) ^c^	64 (24.0)	70 (26.2)	50 (18.7)
Type 2 diabetes (%)	31 (3.7)	15 (48.4) ^b, c^	6 (19.4)	5 (16.1)	5 (16.1)
Prediabetes (%)	270 (31.9)	84 (31.1) ^c^	64 (23.7)	71 (26.3)	51 (18.9)
Postpartum glucose intolerance (%)	301 (35.6)	99 (32.9) ^a, b, c^	70 (23.3)	76 (25.2)	56 (18.6)

*TC* total cholesterol, *GDM* gestational diabetes mellitus, *BMI* body mass index, *GA* gestational age, *GWG* gestational weight gain, *PTB* preterm birth, *OGTT* oral glucose tolerance test, *IFG* impaired fasting glucose, *IGT* impaired glucose tolerance

^*^data are shown as median (IQR) or number (%)

^a, b, c, d, e, f^ Indicates a significant difference between first and second quartile, first and third quartile, first and fourth quartile, second and third quartile, second and fourth quartile, third and fourth quartile, respectively

As shown in Table 1, the median time interval between parturition and postpartum OGTT was 7–8 weeks. The median 2-h BG in the postpartum OGTT increased from 6.4 mmol L^− 1^ in the first TC quartile to 8.6 mmol L^− 1^ in the fourth TC quartile. FBG in the postpartum OGTT of women in the second TC quartile was significantly lower than that of women in the fourth quartile. In addition, 2-h BG in the postpartum period increased with increasing TC quartiles. The overall incidence rates of IFG, IGT and type 2 diabetes were 0.4, 31.6 and 3.7%, respectively. The incidence of any postpartum glucose intolerance, including IFG, IGT and type 2 diabetes, in the first TC quartile (32.9%) was significantly higher than that in the second (23.3%), third (25.2%) and fourth (18.6%) quartiles.

**Fig. 2:**
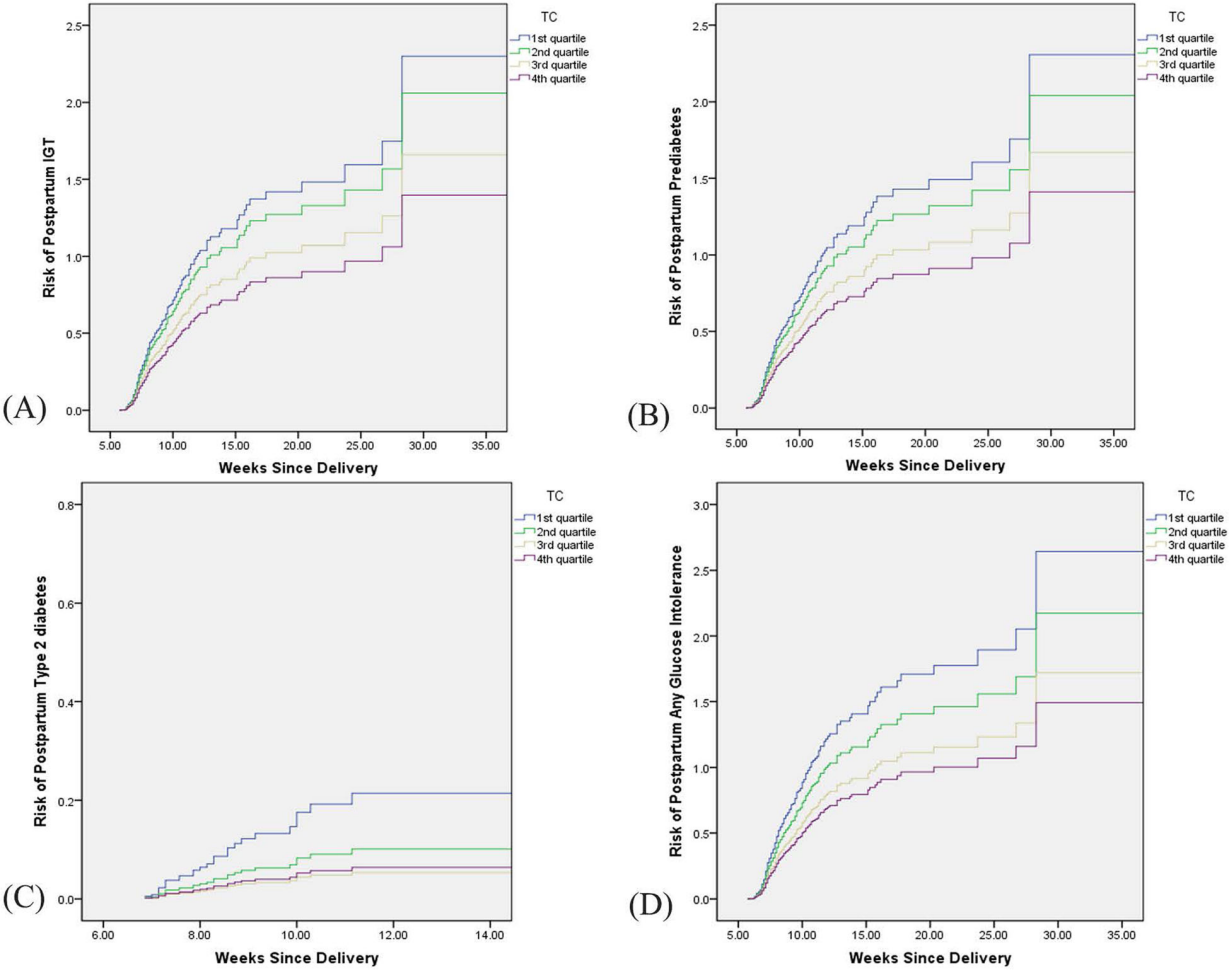
Risk of postpartum IGT (**a**), prediabetes (**b**), type 2 diabetes (**c**) and any glucose intolerance (**d**) with increasing weeks since delivery after GDM pregnancies in different TC quartiles. IGT, impaired glucose tolerance; GDM, gestational diabetes; TC, total cholesterol

After adjusting for maternal age, prepregnancy BMI, time of diagnostic OGTT, time of postpartum OGTT, and the concentrations of TGs, HDL-c, and LDL-c at time of GDM diagnosis, higher TC quartiles remained independently associated with a decreased risk for postpartum aberrant glucose tolerance (Table 2). No significant associations between TGs, HDL-c, and LDL-c and postpartum glucose intolerance were found in this study (Additional file 1). Compared with women in the first TC quartile, women in the second quartile had a decreased risk for IGT, prediabetes, type 2 diabetes and any postpartum abnormal glucose tolerance, but the difference was not significant (Table 2). However, women in the third and fourth TC quartiles had a significantly decreased risk for the abovementioned types of postpartum glucose intolerance compared with women in the first quartile (Table 2). Figure 2 shows the risk of IGT, prediabetes, type 2 diabetes and any postpartum glucose intolerance according to time since delivery in different TC quartiles.

**Table 2:** Hazard Ratio of The Association Between TC Quartiles at the time of GDM Diagnosis and Risk of Postpartum Impaired Glucose Tolerance (*N* = 845)

		N (%)	HR (95%CI)	*P*	Adjusted HR (95%CI)^a^	*P*
IGT	First	83 (30.6)	Reference		Reference	
	Second	64 (23.6)	0.875 (0.631, 1.214)	0.423	0.892 (0.642, 1.239)	0.494
	Third	70 (25.9)	0.704 (0.512, 0.968)	0.031	0.703 (0.510, 0.971)	0.032
	Fourth	54 (19.9)	0.562 (0.395, 0.799)	0.001	0.569 (0.400, 0.811)	0.002
Prediabetes^b^	First	84 (30.7)	Reference		Reference	
	Second	64 (23.4)	0.864 (0.623, 1.197)	0.380	0.880 (0.634, 1.222)	0.446
	Third	71 (25.9)	0.705 (0.514, 0.968)	0.031	0.705 (0.512, 0.971)	0.032
	Fourth	55 (20.0)	0.566 (0.400, 0.803)	0.001	0.574 (0.404, 0.815)	0.002
Type 2 diabetes	First	15 (48.4)	Reference		Reference	
	Second	6 (19.4)	0.457 (0.177, 1.181)	0.106	0.455 (0.176, 1.174)	0.103
	Third	5 (16.1)	0.242 (0.085, 0.687)	0.008	0.239 (0.084, 0.680)	0.007
	Fourth	5 (16.1)	0.296 (0.106, 0.825)	0.020	0.293 (0.105, 0.817)	0.019
Postpartum glucose intolerance^c^	First	99 (32.9)	Reference		Reference	
	Second	70 (23.3)	0.802 (0.590, 1.091)	0.802	0.814 (0.598, 1.109)	0.192
	Third	76 (25.2)	0.634 (0.470, 0.856)	0.003	0.632 (0.467, 0.855)	0.003
	Fourth	56 (18.6)	0.526 (0.379, 0.731)	0.000	0.531 (0.382, 0.739)	0.000

*TC* total cholesterol, *GDM* gestational diabetes mellitus, *HR* hazard ratio, *CI* confidential interval, *IGT* impaired glucose tolerance

^a^adjusted for maternal age, pre-pregnancy BMI, time of diagnostic OGTT, time of postpartum OGTT, the concentrations of TG, HDL-c, LDL-c at time of GDM diagnosis and insulin treatment or not

^b^including both IFG and IGT

^c^including IFG, IGT and type 2 diabetes

## Discussion

This study found that the overall incidence rates of IFG, IGT and type 2 diabetes were 0.4, 31.6, and 3.7% in women with recent GDM, respectively. The most interesting finding of this study was that a higher TC quartile at diagnosis of GDM was associated with a decreased risk of postpartum impaired glucose tolerance. Women in the highest TC quartile (>7.0 mmol L^− 1^) had approximately half the risk of developing any type postpartum glucose intolerance compared with women in the lowest TC quartile (<5.5 mmol L^− 1^).

To the best of found knowledge, no study to date has investigated the relationship between TC at GDM diagnosis and postpartum disorders of glucose metabolism. Lappas et al. found that postnatal TC was significantly higher in women who developed type 2 diabetes after GDM pregnancies than in those who did not when they investigated whether serum lipids were associated with the risk of type 2 diabetes via lipidomics [17]. Katon et al. found that higher HbA1c at GDM diagnosis was associated with an increased risk of disorders of glucose metabolism [18]. Another study suggested that an HDL-c level less than 50 mg dl^− 1^ at the time of GDM diagnosis and age older than 35 years were the best predictors of the development of type 2 diabetes after GDM [12]. Another study demonstrated that women whose FBG was ≥5.4 mmol L^− 1^, 2-h BG was ≥9.3 mmol L^− 1^ at the time of GDM diagnosis, or who had a history of polycystic ovary syndrome were at higher risk of developing glucose intolerance [19].

The findings of this study contradicted the common idea that TC is always recognized as an independent risk factor for metabolic syndrome, including diabetes and coronary heart diseases [20]. A possible reason is the deficiency of cholesterol synthesis. To meet the requirements of fetal growth and development, the synthesis of TC substantially increases during later pregnancy [21]. Synthesis of TC starts with the mevalonate pathway [22]. Three molecules of acetyl CoA form 3-hydroxy-3-methylglutaryl CoA (HMG-CoA) via two condensations. Then, HMG-CoA is reduced to mevalonate by HMG-CoA reductase (HMGCR). HMGCR is the rate-limiting enzyme because the production of mevalonate is the irreversible step in cholesterol synthesis. Mevalonate is finally converted to cholesterol through a 25-step process. In addition, the biosynthesis of cholesterol is directly regulated by the current level of cholesterol, and the main regulatory mechanism is the sensitivity of the endoplasmic reticulum to intracellular cholesterol, which is regulated by sterol regulatory element-binding transcription protein (SREBP). Pendzialek et al. found that the expression of HMGCR and SREBP was reduced in diabetic rabbits [23]. Thus, the decreased expression of HMGCR and SREBP, which caused the relatively low TC level, was speculated to be involved in the development and progression of diabetes mellitus. In addition, cholesterol acts as a precursor to many hormones, such as steroid hormones. Previous studies suggested that estrogen deficiency might contribute to obesity, type 2 diabetes and cardiovascular diseases [24]. Lassance et al. [25] found that serum concentrations of estradiol and progesterone were significantly lower in obese pregnant women (BMI 30–35 kg m^− 2^) than in normal-weight pregnant women (BMI 19–25 kg m^− 2^). In this study, we assumed that the concentration of TC in women in the lower TC quartile did not meet the required TC level due to the reduction in HMGCR or SREBP, which finally caused the occurrence of prediabetes and diabetes after pregnancy. In addition, contrary to the well-recognized role of TC in cardiovascular disease, several studies have shown a negative relationship between the level of TC and mortality [26, 27]. Anderson et al. found an 11% increase in overall mortality and a 14% increase in cardiovascular disease-associated mortality with a reduction in TC of 1 mg dL ^− 1^ per year [26]. In the Platelet glycoprotein IIb/IIIa in Unstable angina: Receptor Suppression Using Integrilin Therapy trial, hypercholesterolemia was protective in patients with non-ST segment elevation acute coronary syndromes [28]. The reason hypercholesterolemia was related to better outcomes in patients with cardiovascular diseases remains unclear. Wang et al. [27] attributed the paradox phenomenon to the fact that patients diagnosed with hypercholesterolemia may have more medical contact, such as receiving statin treatment. In this study, none of the pregnant women received cholesterol-lowering treatment due to the lack of recommended TC levels in pregnant women. Nevertheless, women whose serum concentration of TC was abnormally increased might voluntarily adjust their lifestyle, which might improve postpartum outcomes to some extent. We thus hypothesized that the increase in serum TC might be a compensatory and adaptive response to pregnancy. Failure to compensate or deficiency of compensation may be a pathological condition that finally generates disturbances in postnatal glucose metabolism. However, excessively increased TC was generally considered an independent risk factor for pregnancy complications. The “fetal origins hypothesis” suggested that the offspring of women with hypercholesterolemia during pregnancy might have an increased risk of cardiovascular disease later in life [29]. Conversely, previous studies reported that a decreased concentration of TC was associated with an enhanced risk for fetal growth restriction and PTB [30, 31]. Therefore, the association between TC and postpartum impaired glucose tolerance and the optimal level of maternal TC during pregnancy warrants further investigation.

Previous studies investigated the incidence of disorders of glucose metabolism in the early postpartum period after recent GDM. Ingram et al. found that 11.1% (13/117) and 8.5% (10/117) of GDM women who underwent OGTT at 6–12 weeks postpartum suffered IFG and IGT, respectively, and none of the women had overt diabetes in a study conducted in Australia [19]. Carmody et al. reported that 5.7% (67/1149), 9.0% (104/1149) and 3.8% (43/1149) of women who had a history of GDM developed IFG, IGT and type 2 diabetes, respectively, from January 2008 to December 2012 in a study conducted in Ireland [32]. Another study conducted by McClean et al. reported that the incidence rates of IFG, IGT and DM at 6 weeks postpartum were 5.0% (49/985), 11.6% (114/985) and 11.1% (109/985), respectively, in the UK [33]. Capula et al. found that 32.1% (146/454) of women had prediabetes (IFG and IGT) and 4.0% (18/454) had type 2 diabetes in the early postpartum period among women with previous GDM in Italy [34]. However, the incidence of IFG in this study was 0.4%, which was significantly lower than that in previous studies. Weyer et al. found that women with isolated IFG and combined IFG/IGT were heavier than those with isolated IGT [35]. The possible reason this study’s results were inconsistent with previous studies is that the proportion of obesity among fertile women in China, especially in Southern China, is lower than that in Western countries, and normal-weight and underweight women are at low risk of developing IFG.

The prepregnancy BMI and the incidence of cesarean delivery of women in the first TC quartile were also found to be significantly higher than those among women in the higher TC quartile. Given that increased risks of GDM, type 2 diabetes, and cesarean delivery were associated with increasing BMI, we thus hypothesized that the increase in serum TC, to some extent, had a compensatory and protective response to pregnancy. Failure to compensate may lead to pregnancy complications, adverse pregnancy outcomes and even postnatal complications. However, the underlying mechanism still needs further study.

In addition, only 30.1% of the included women returned to the hospital for OGTT at 6–12 weeks postpartum in the study. The adherence rate was far lower than that in previous studies, in which the rates were 45–70.9% [19, 36–38]. One of the possible reasons was that residences of participants were too far from the hospital to return for OGTT easily. Other reasons included the low level of income and education, lack of antenatal care, and inadequate health insurance. Due to the increasing diabetes-associated health and economic burden, effective measures, including health education and reinforced antenatal care, need to be implemented to reduce the incidence of subsequent diabetes in the future.

### Study strengths and limitations

The strengths of this study included the sample size and the detailed data about demographic, medical and obstetric history. In addition, prospective measurement of the relationship between TC quartiles at GDM diagnosis and impaired postnatal glucose tolerance was another strength of the study.

Nevertheless, there still exist several limitations in this study. When analyzing the relationship between increasing TC quartile and the risk of postpartum impaired glucose tolerance, the time scale used was weeks since delivery. However, women who were diagnosed with IFG, IGT or type 2 diabetes at the time of postpartum OGTT can have developed glucose intolerance at any time between delivery and postpartum OGTT, even during pregnancy. Further study is needed to address this problem by increasing the frequency of glucose monitoring and the time of follow-up. Another limitation is that we were unable to collect information, including income, education level, residence and lifestyle habits, which may influence these results. Moreover, the applicability of these results may be limited because pregnant women from one regional university hospital may not be reflective of all pregnant Chinese women; data from a multicenter trial would be more representative. Additionally, subgroup analysis is necessary to clarify different cholesterol particles, including HDL-c and LDL-c, in future studies.

## Conclusions

In conclusion, compared with women in the first TC quartile at the time of GDM diagnosis, women in higher TC quartiles were found to have a decreased risk for glucose intolerance. Thus, excessively low TC might lead to adverse postpartum outcomes. Excessively increased TC, however, has been extensively shown to be an independent risk factor for pregnancy and postpartum complications. Therefore, further studies on the optimal concentration of maternal serum TC throughout pregnancy will be of importance, considering that both high and low TC levels would result in adverse pregnancy and postpartum outcomes. Controlling the maternal TC level to the appropriate level might improve adverse maternal and fetal complications in both the short term and long term and reduce the associated economic burden.

## Supplementary information

**Additional file 1: Table S1.** Hazard Ratio of The Association Between TG Quartiles at the time of GDM Diagnosis and Risk of Postpartum Impaired Glucose Tolerance (*N* = 845). **Table S2.** Hazard Ratio of The Association Between HDL-c Quartiles at the time of GDM Diagnosis and Risk of Postpartum Impaired Glucose Tolerance (*N*=845). **Table S3.** Hazard Ratio of The Association Between LDL-c Quartiles at the time of GDM Diagnosis and Risk of Postpartum Impaired Glucose Tolerance (*N* = 845).

## Data Availability

The datasets generated and/or analyzed during the current study were available from the corresponding author on reasonable request.

## Abbreviations

GDM: Gestational diabetes mellitus
IFG: Impaired fasting glucose
IGT: Impaired glucose tolerance
TC: Total cholesterol
LDL: Low-density lipoprotein
HDL: High-density lipoprotein
OGTT: Oral glucose tolerance test
BMI: Body mass index
PGDM: Pre-gestational diabetes mellitus
PTB: Preterm birth
FBG: Fasting blood glucose
HR: Hazard ratio
HMGCR: HMG-CoA reductase
SREBP: Sterol regulatory element binding transcription protein
GA: Gestational age
