# Supplementary material for: The relationship between total cholesterol and postpartum impaired glucose tolerance in women with gestational diabetes mellitus
Source: Lipids Health Dis. 2020 Jun 18;19:142. doi: 10.1186/s12944-020-01316-5 (PMC7302143; doi:10.1186/s12944-020-01316-5)
Supplement: Supplementary file 1 — Additional file 1: Table S1. Hazard Ratio of The Association Between TG Quartiles at the time of GDM Diagnosis and Risk of Postpartum Impaired Glucose Tolerance (N = 845). Table S2. Hazard Ratio of The Association Between HDL-c Quartiles at the time of GDM Diagnosis and Risk of Postpartum Impaired Glucose Tolerance (N=845). Table S3. Hazard Ratio of The Association Between LDL-c Quartiles at the time of GDM Diagnosis and Risk of Postpartum Impaired Glucose Tolerance (N = 845). [file 12944_2020_1316_MOESM1_ESM.docx]

Supplementary Table 1. Hazard Ratio of The Association Between TG Quartiles at the time of GDM Diagnosis and Risk of Postpartum Impaired Glucose Tolerance (N=845)

|  |  | N (%) | HR (95%CI) | *P* |
| --- | --- | --- | --- | --- |
| IGT | First | 56 (20.7) | Reference |  |
|  | Second | 75 (27.7) | 1.299 (0.918, 1.839) | 0.140 |
|  | Third | 67 (24.7) | 1.160 (0.813, 1.655) | 0.413 |
|  | Fourth | 73 (26.9) | 1.306 (0.917, 1.858) | 0.139 |
| Prediabetes ^#^ | First | 56 (20.4) | Reference |  |
|  | Second | 75 (27.4) | 1.299 (0.918, 1.839) | 0.140 |
|  | Third | 69 (25.2) | 1.194 (0.839, 1.700) | 0.324 |
|  | Fourth | 74 (27.0) | 1.324 (0.932, 1.883) | 0.117 |
| Type 2 diabetes | First | 7 (22.6) | Reference |  |
|  | Second | 10 (32.3) | 1.353 (0.512, 3.576) | 0.542 |
|  | Third | 9 (29.0) | 1.266 (0.471, 3.402) | 0.640 |
|  | Fourth | 5 (16.1) | 0.697 (0.217, 2.239) | 0.545 |
| Postpartum glucose intolerance ^†^ | First | 63 (20.7) | Reference |  |
|  | Second | 85 (27.9) | 1.307 (0.942, 1.813) | 0.109 |
|  | Third | 78 (25.6) | 1.202 (0.862, 1.676) | 0.279 |
|  | Fourth | 79 (25.8) | 1.254 (0.896, 1.754) | 0.187 |

TC, total cholesterol; GDM, gestational diabetes mellitus; HR, hazard ratio; CI, confidential interval; IGT, impaired glucose tolerance.

# including both IFG and IGT.

† including IFG, IGT and type 2 diabetes.

Supplementary Table 2. Hazard Ratio of The Association Between HDL-c Quartiles at the time of GDM Diagnosis and Risk of Postpartum Impaired Glucose Tolerance (N=845)

|  |  | N (%) | HR (95%CI) | *P* |
| --- | --- | --- | --- | --- |
| IGT | First | 66 (24.4) | Reference |  |
|  | Second | 66 (24.4) | 0.996 (0.706, 1.404) | 0.980 |
|  | Third | 76 (28.0) | 1.013 (0.728, 1.410) | 0.940 |
|  | Fourth | 63 (23.2) | 0.834 (0.588, 1.184) | 0.311 |
| Prediabetes ^#^ | First | 67 (24.5) | Reference |  |
|  | Second | 68 (24.8) | 1.010 (0.719, 1.418) | 0.954 |
|  | Third | 76 (27.7) | 0.998 (0.718, 1.387) | 0.898 |
|  | Fourth | 63 (23.0) | 0.822 (0.580, 1.165) | 0.270 |
| Type 2 diabetes | First | 11 (35.5) | Reference |  |
|  | Second | 9 (29.0) | 0.837 (0.345, 2.033) | 0.695 |
|  | Third | 7 (22.6) | 0.552 (0.214, 1.425) | 0.220 |
|  | Fourth | 4 (12.9) | 0.336 (0.107, 1.057) | 0.062 |
| Postpartum glucose intolerance ^†^ | First | 78 (25.6) | Reference |  |
|  | Second | 77 (25.2) | 0.985 (0.718, 1.353) | 0.927 |
|  | Third | 83 (27.2) | 0.934 (0.685, 1.274) | 0.667 |
|  | Fourth | 67 (22.0) | 0.753 (0.541, 1.049) | 0.093 |

TC, total cholesterol; GDM, gestational diabetes mellitus; HR, hazard ratio; CI, confidential interval; IGT, impaired glucose tolerance.

# including both IFG and IGT.

† including IFG, IGT and type 2 diabetes.

Supplementary Table 3. Hazard Ratio of The Association Between LDL-c Quartiles at the time of GDM Diagnosis and Risk of Postpartum Impaired Glucose Tolerance (N=845)

|  |  | N (%) | HR (95%CI) | *P* |
| --- | --- | --- | --- | --- |
| IGT | First | 78 (28.8) | Reference |  |
|  | Second | 70 (25.8) | 1.061 (0.765, 1.471) | 0.722 |
|  | Third | 70 (25.8) | 0.840 (0.607, 1.163) | 0.293 |
|  | Fourth | 53 (19.6) | 0.720 (0.503, 1.030) | 0.072 |
| Prediabetes ^#^ | First | 79 (28.8) | Reference |  |
|  | Second | 71 (25.9) | 1.061 (0.767, 1.468) | 0.720 |
|  | Third | 70 (25.5) | 0.828 (0.599, 1.146) | 0.255 |
|  | Fourth | 54 (19.8) | 0.724 (0.508, 1.032) | 0.074 |
| Type 2 diabetes | First | 14 (45.2) | Reference |  |
|  | Second | 4 (12.9) | 0.350 (0.114, 1.068) | 0.065 |
|  | Third | 6 (19.4) | 0.385 (0.148, 1.005) | 0.051 |
|  | Fourth | 7 (22.5) | 0.552 (0.222, 1.372) | 0.201 |
| Postpartum glucose intolerance ^†^ | First | 93 (30.5) | Reference |  |
|  | Second | 75 (24.6) | 0.955 (0.702, 1.298) | 0.768 |
|  | Third | 76 (24.9) | 0.760 (0.560, 1.031) | 0.078 |
|  | Fourth | 61 (20.0) | 0.697 (0.501, 0.970) | 0.032 |

TC, total cholesterol; GDM, gestational diabetes mellitus; HR, hazard ratio; CI, confidential interval; IGT, impaired glucose tolerance.

# including both IFG and IGT.

† including IFG, IGT and type 2 diabetes.
